# EttA is likely non-essential in *Staphylococcus aureus* persistence, fitness or resistance to antibiotics

**DOI:** 10.1186/s12866-020-01970-w

**Published:** 2020-09-17

**Authors:** Michal Meir, Anna Rozenblit, Simona Fliger, Yuval Geffen, Daniel Barkan

**Affiliations:** 1 The Ruth Rappaport Children’s Hospital, Rambam Health Care Campus, Haifa, Israel; 2 Clinical Microbiology Laboratory, Rambam Health Care Campus, Haifa, Israel; 3 Koret School of Veterinary Medicine, The Robert H. Smith Faculty of Agriculture, Food and Environment, The Hebrew University of Jerusalem, Rehovot, Israel

**Keywords:** *Staphylococcus aureus*, Antibiotic resistance, Antibiotic tolerance, Persistence, Fitness, Killing curve, Ribosomal hibernation

## Abstract

**Background:**

Tolerance to antibiotics and persistence are associated with antibiotic treatment failures, chronic-relapsing infections, and emerging antibiotic resistance in various bacteria, including *Staphylococcus aureus*. Mechanisms of persistence are largely unknown, yet have been linked to physiology under low-ATP conditions and the metabolic-inactive state. EttA is an ATP-binding cassette protein, linked in *Eschrechia coli* to ribosomal hibernation and fitness in stationary growth phase, yet its role in *S. aureus* physiology is unknown.

**Results:**

Using whole genome sequencing (WGS) of serial clinical isolates, we identified an EttA-negative *S. aureus* mutant (*ettA*^*stop*^), and its isogenic wild-type counterpart. We used these two isogenic clones to investigate the role of *ettA* in *S. aureus* physiology in starvation and antibiotic stress, and test its role in persistence and antibiotic tolerance. *ettA*^*stop*^ and its WT counterpart were similar in their antibiotic resistance profiles to multiple antibiotics. Population dynamics of *ettA*^*stop*^ and the WT were similar in low-nutrient setting, with similar recovery from stationary growth phase or starvation. Supra-bacteriocidal concentration of cefazolin had the same killing effect on *ettA*^*stop*^ and WT populations, with no difference in persister formation.

**Conclusions:**

Lack of *ettA* does not affect *S. aureus* antibiotic resistance, beta-lactam tolerance, resilience to starvation or fitness following starvation. We conclude the role of *ettA* in *S. aureus* physiology is limited or redundant with another, unidentified gene. WGS of serial clinical isolates may enable investigation of other single genes involved in *S. aureus* virulence, and specifically persister cell formation.

## Background

*Staphylococcus aureus* is a major human pathogen, responsible for a variety of acute, as well as chronic and relapsing infections such as osteomyelitis and endocarditis and is notorious for its ability to form biofilm on implanted devices. Persisters are dormant phenotypic variants of bacterial cells that are tolerant to killing by antibiotics, and are associated with chronic infections and antibiotic treatment failures [1]. It has been recently reported that emergence of persister *S. aureus* bacteria in a patient receiving seemingly adequate treatment, precedes and facilitates the emergence of true resistant bacteria [2]. While mechanisms of persistence appear to be heterogeneous and mostly poorly characterized, it appears that many of them involve translation mechanisms and slowing or inhibiting RNA translation into proteins [3, 4]. Specifically in *S. aureus*, persistence has been linked to lower ATP levels and stationary growth phase [5]. Elucidating the genetic basis of persistence and regulation of protein synthesis under antibiotic or metabolic stress is of importance in developing strategies to combat emerging antibiotic resistance, and for developing novel therapeutics for chronic infections.

Energy-dependent translational throttle protein (EttA) is an ATP-binding cassette family (ABC-F) protein. Unlike other ABC-F proteins in gram positive bacteria, known to mediates resistance to ribosome-active antibiotics [6], EttA does not confer antibiotic resistance, but functions as a translation factor to limit ribosomal activity in response to low ATP levels [6–8]. In *Eschrechia coli*, expression of EttA increases in stationary phase allowing a hibernation state of low ribosomal translation, until ATP levels are restored [9, 10]. No resistance phenotype was found in a targeted deletion mutant in *E. coli*, yet a functional EttA was important for rapid emergence from stationary phase [9]. The role of EttA in *S. aureus* physiology has not been elucidated. Considering the role of EttA in mediating hibernation state in *E. coli*, we hypothesized EttA may have a similar role in *S. aureus*. Furthermore, considering the link between persistence in *S. aureus* and stationary growth phase dynamics, [5] we hypothesized that EttA in *S. aureus* may be involved in antibiotic tolerance, resistance to nutrient depletion, and emergence from stationary growth phase. We tested these hypotheses using an *ettA*-negative *S. aureus* clinical mutant, comparing it to its isogenic *ettA* wild-type strain.

## Results

### Isolation of an EttA-negative *S. aureus* mutant and its isogenic *wild-type* strain

We used *S. aureus* strains isolated from serial blood cultures during a continuous *S. aureus* bacteremia. Blood cultures obtained on four sequential days grew *S. aureus*, despite early beta-lactam and glycopeptide-based antimicrobial therapy confirmed to be microbiologically appropriate, and removal of the catheter. Whole genome sequencing (WGS) was performed on four isolates, each from a different day of bacteremia, and sequences were aligned searching for genetic variations. Analysis revealed the strains to be of the same clone, given scant genetic variations if any. All strains were negative for SCCmec. Specifically, the strain isolated on day 1 of bacteremia was found to have a single nucleotide change (C= > T at position 490) in the gene *ettA*, causing a premature stop codon (TAA) after 163 of 628 amino acids in total. The point mutation was verified using PCR and Sanger sequencing. No other mutations were found in this clone, *ettA*^stop^, as compared to the other WT *S. aureus* clones isolated from this patient. By comparing the WT clinical *S. aureus* isolate to its EttA-depleted mutant (*ettA*^stop^), we could examine the role of *ettA* in *S. aureus* antibiotic resistance, persistence, response to nutrient depletion and emergence from stationary phase.

### Lack of EttA does not affect susceptibility to antibiotics

We determined the minimal inhibitory concentration (MIC) to several commonly used anti-staphylococcal antibiotics in the *ettA*^stop^ mutant and its WT *S. aureus* counterpart, by broth dilution assay, using both Luria broth (LB) and Mueller-Hinton (MH) media. Given the possible role of ABC Family proteins in conferring resistance to ribosomal-active antibiotics [8], we tested MICs of both strains to 50s- and 30s-binding antibiotics, as well as to other classes of antibiotics, including beta-lactams. We found no difference in the MIC values for cefazolin, gentamicin, azithromycin, vancomycin, or ciprofloxacin. Linezolid and erythromycin had a single (1:2) dilution differences in LB and MH accordingly (Table 1).

**Table 1:**
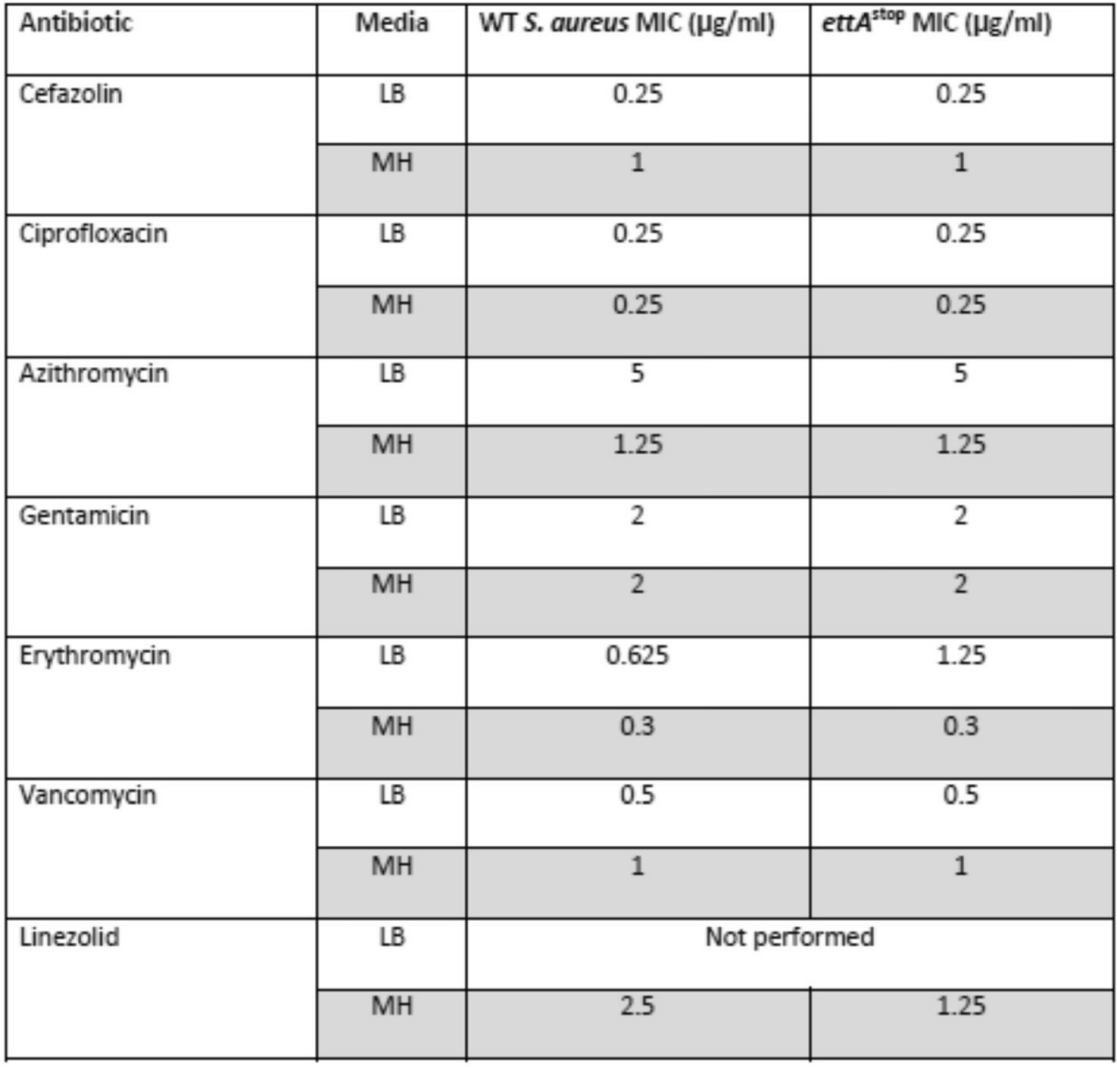
The *ettA*^stop^ mutant and its WT counterpart had similar antibiotic-resistance profiles to various antibiotics, including ribosomal-active drugs. MIC was determined by broth dilution, in two different growth media. LB – Luria Broth, MH – Mueller Hinton media

### *ettA*^*stop*^ and WT *S. aureus* have similar population dynamics following starvation

**Fig. 1:**
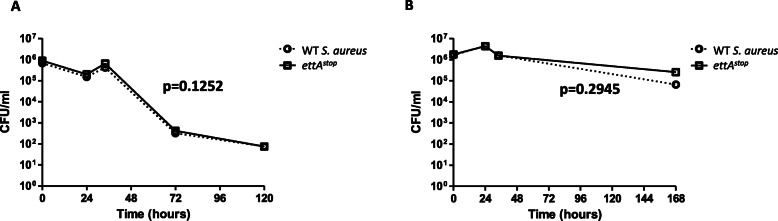
WT *S. aureus* and *ettA*^*stop*^ are similarly resilient to nutrient starvation in two starvation models. **a** – Bacteria were grown to stationary phase then diluted 1:100 prior to starvation in PBS. PBS Broth was plated in triplicates for CFU counts at timely intervals as shown. **b** – Bacteria were grown to stationary phase in LB. LB broth was plated in triplicates for CFU at timely intervals to determine survival. Points on graphs represent mean values of CFU/ml at each time points. Paired t-test was performed to demonstrate differences between strains, and was found non-significant (*p* value shown)

Considering the possible role of *ettA* in ATP-depleted conditions [9], we sought to compare the resilience of WT *S. aureus* and *ettA*^*stop*^ to nutrient depletion or cell-starvation. We used two starvation models. In the first model, each strain was grown in LB to an OD_600_ of 1 (stationary phase), then diluted 1:100 into nutrient-depleted media (PBS), and left at 37 °C. Bacterial survival was determined by CFU count. Survival of the WT *S. aureus* and *ettA*^*stop*^ was similar (non-significant paired t-test, t = 1.934, df = 4, *p*-value 0.1252) (Fig. 1a). In the second model, we compared the survival of WT *S. aureus* and *ettA*^*stop*^ mutant in a nutrient-limited environment. Each strain was grown in LB to stationary phase, left in the same broth, and plated for CFU count at timely intervals. CFU counts of both strains were identical in the first 48 h, with a non-significant advantage of *ettA*^*stop*^ after 168 h (non-significant paired t-test, t = 1.267 df = 3, p- value = 0.2945) (Fig. 1b).

### Lack of EttA does not affect antibiotic killing dynamics

**Fig. 2:**
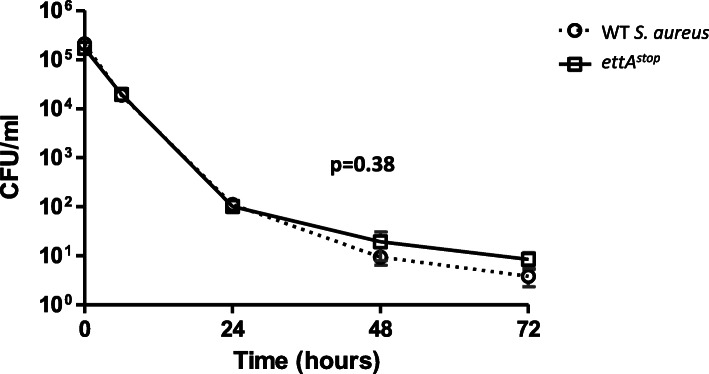
WT *S. aureus* and *ettA*^*stop*^ demonstrate similar beta-lactam–induced killing dynamics. Cefazolin 16XMIC was added to WT and *ettA*^*stop*^ *S. aureus* cultures grown to OD600 of 0.2. Broths (sextuplets) were plated for CFU counts at timely intervals. Graph shows mean and SEM for each time point. Paired t-test noted non-significant difference between strains (*p* = 0.38). Similar results were noted in repeated experiments

To examine if a functional EttA affect the ability of *S. aureus* to form antibiotic persister cells, we evaluated in-vitro persister formation in response to cefazolin, as a representative cell-wall active drug. Strains were grown to exponential phase (OD_600_ 0.2). Cefazolin was then added to each broth to reach supra-bactericidal concentrations (16XMIC). Broths were plated for CFU at timely intervals. A representative experiment is shown in Fig. 2. Killing dynamics of WT *S. aureus* and *ettA*^*stop*^ were similar; paired t-test noted non-significant difference between strains (*p* = 0.38).

### Lack of EttA does not impair *S. aureus* fitness in nutrient-depleted environments

**Fig. 3:**
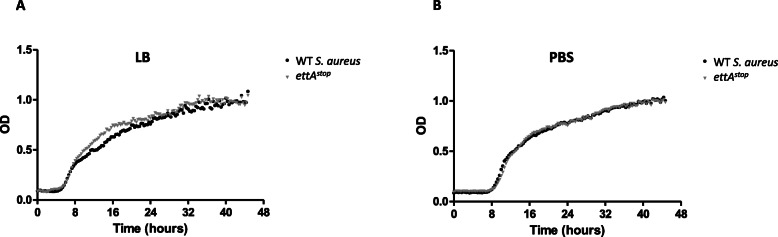
WT *S. aureus* and *ettA*^*stop*^ have similar growth-recovery dynamics. **a** - Strains were grown to stationary phase in LB for over 144 h, diluted to 10^6^ CFU/ml and immediately regrown in LB. **b**- Strains were grown in a nutrient depleted environment (PBS) for 72 h. Strains were then diluted to 10^3^ CFU/ml and regrown in LB. In both experiments OD_600_ was measured in sextuplets every 20 min using a microplate reader. Graphic data points show mean and SEM for each measurement

It was previously shown that in *E. coli*, *ettA* deletion caused decreased fitness, manifesting as slower recovery from stationary phase [9]. In order to examine the effect of *ettA* on fitness in *S. aureus,* we evaluated growth-recovery dynamics in two nutrient-depletion models. In the first model, WT and the *ettA*^stop^ strains were inoculated into LB, grown to OD_600_ 1, then continuously grown in the same media for an additional 144 h. In the second model, strains were grown to exponential phase, and then diluted into nutrient-free media (PBS) for additional 72 h. Following nutrient-depletion, bacteria in both models were diluted into pre-warmed fresh LB, and immediately allowed to re-grow in sextuplets using a 96 well plate. Growth dynamics were recorded by a microplate reader (Spectramax i3), using serial OD_600_ measurements every 20 min, for 48 h. In both models, time to recovery (exponential growth phase), peak concentration (OD_600_ at stationary phase) and time to reaching stationary phase were similar in WT *S. aureus* and *ettA*^*stop*^ (Fig. 3).

## Discussion

Persister bacteria pose clinical challenges including relapsing chronic infections, and antibiotic treatment failures. The formation of persisters facilitates the emergence of true resistant bacteria [2]. The elucidation of the molecular events leading to persister formation may help us counter these events. *S. aureus* is a common, highly virulent human and animal pathogen, causing severe protracted sub-chronic and chronic infections. Genetic manipulation of *S. aureus* is challenging due to a strong restriction barrier. Despite recent advancements in genetic manipulation [11], transformation and gene-deletion in *S. aureus* remain relatively complex, requiring specialized plasmids. These difficulties make the investigation of the role of specific *S. aureus* genes challenging, contributing to a knowledge gap in the field. EttA belongs to the ABC protein family, associated with resistance to ribosomal-active antibiotics [6, 8]. In *E. coli*, *ettA* is involved in ribosome hibernation, cell fitness and emergence from stationary phase [8–10], possibly linking it to persistent qualities. In *S. aureus*, transposon-mutant library analyses have shown *ettA* to be a non-essential gene [12, 13]. In a study published in 2014, the transcriptome of *S. aureus* was examined under various conditions, including antibiotic challenge in several different media [14]. We examined the expression of *ettA* in this study, but found no consistent changes that would point to a specific role.

Here we encountered an opportunity to characterize the role of *ettA* in *S. aureus* using *ettA*^*stop*^, a mutant lacking a functional EttA protein due to a nonsense mutation at the first third of the gene. Surprisingly, we found that *ettA*^*stop*^ was not different from its WT clone in bacterial growth, resilience to starvation and antibiotic resistance and persistence. It is possible that a distinct phenotype of this mutant would be evident under specific physiologic conditions. However, given the similarities shown above, we evaluate the role *ettA* plays in staphylococcal pathogenesis to be limited. It remains to be determined whether *ettA* is dispensable in *S. aureus* ribosome physiology, or that its functions are shared or overlapped by another gene, making *ettA* redundant. Although we cannot determine which is the correct possibility, our report concerns specifically the role of *ettA* [protein WP_000525111.1, gene 00729 in *Staphylococcus aureus subsp. aureus* NCTC 8325 (nucleotides 712,364–714,247, positive strand)] and for this we believe the evidence provided here are unequivocal.

Our study also demonstrates that as WGS technology becomes less expensive and more accessible, valuable information can be discovered by judicial sequencing and analysis of clinically-obtained strains. Genetic examination of *S. aureus* strains isolated in prolonged bacteremias, reactivation of chronic infections or antibiotic failures may specifically elucidate persistence mechanisms.

## Conclusions

We have examined and shown the role of the *ettA* gene in staphylococcal physiology, and possibly virulence. Our results suggest this role is likely limited, in terms of persistence, antibiotic resistance and fitness. Our study also exemplifies the rich possibilities in staphylococcal virulence and physiology research that can be achieved via systematic whole genome sequencing of clinical isolates, especially of out-of-the ordinary clinical scenarios.

## Methods

### Growth and MIC determination

bacteria were grown in LB media. MIC was determined by inoculation of 10^3^ bacterial colony forming units into 10 ml of LB or Mueller-Hinton (MH) media, with serial dilutions of the tested antibiotic. The MIC was determined as the concentration with no apparent growth 12 h after a control tube with no antibiotics became turbid.

### Isolation of the *ettA*^stop^ mutant

a single colony from blood-agar plates used in the clinical microbiology lab, from four consecutive days of bacteremia, were picked and grown in LB. genomic DNA was extracted and sent for whole-genome sequencing (WGS) on an illumina platform. After establishing one of the clones was mutated in the *ettA* gene, other clones from the same day were examined, and found to be of WT genotype. We therefore concluded the mutant clone was a unique mutation, not representing the whole population. For confirmation, PCR of the presumed mutated area was performed, using primers 250 bp up- and down-stream of the mutation (5′–ACTTCTCTTTCAGCGCGCATTTCAA–3′; and 5′– GATGCAGTATTAAGTTCTGATAC–3′). The PCR product was sent for Sanger sequencing, and the mutation was confirmed.

### Antibiotic killing curves

Bacteria were grown overnight in LB, re-diluted into fresh LB to an OD_600_ of 0.01, and allowed to grow to an O.D_600_ of 0.2 (total volume of 10 ml). This was done to insure all bacteria are in exponential phase. At this point, bacteria were plated for CFU determination (T = 0), and cefazolin was added to a concentration 16 times that of the MIC. Bacteria were plated in triplicates at timely intervals during the first 24 h to determine killing dynamics, and again at 24 and 48 h to determine the number of persister bacteria.

### Whole genome sequencing of clinical strains

Library preparation, sequencing, and mapping of sequence data and variant calling were performed by the Genome Research Core (GRC) at the University of Illinois at Chicago (UIC). Coverage was approximately 98% of the genome, with X250 coverage (~1Gb of data total per genome).

## Data Availability

the datasets used and analyzed, as well as the bacterial strains, are available from the corresponding author on reasonable request.

## Abbreviations

LB: Luria Broth
MH: Mueller-Hinton Broth
WT: Wild type
ATP: Adenosine-triPhosphate
MIC: Minimal Inhibitory Concentration
WGS: Whole Genome Sequencing
OD: Optical Density

## References

[1] Allison KR, Brynildsen MP, Collins JJ (2011). Metabolite-enabled eradication of bacterial persisters by aminoglycosides. Nature..

[2] Liu J, Gefen O, Ronin I, Bar-Meir M, Balaban NQ (2020). Effect of tolerance on the evolution of antibiotic resistance under drug combinations. Science..

[3] Shah D, Zhang Z, Khodursky AB, Kaldalu N, Kurg K, Lewis K (2006). Persisters: a distinct physiological state of *E. coli*. BMC Microbiol.

[4] Gefen O, Gabay C, Mumcuoglu M, Engel G, Balaban NQ (2008). Single-cell protein induction dynamics reveals a period of vulnerability to antibiotics in persister bacteria. Proc Natl Acad Sci.

[5] Conlon B, Rowe S, Brown A (2016). Persister formation in Staphylococcus aureus is associated with ATP depletion. Nat Microbiol.

[6] Sharkey LKR, Edwards TA, O’Neill AJ (2016). ABC-F proteins mediate antibiotic resistance through ribosomal protection. Wright GD, ed. MBio..

[7] Fredrick K, Ibba M (2014). The ABCs of the ribosome. Nat Struct Mol Biol.

[8] Murina V, Kasari M, Takada H (2019). ABCF ATPases involved in protein synthesis, ribosome assembly and antibiotic resistance: structural and functional diversification across the tree of life. J Mol Biol.

[9] Boel G, Smith PC, Ning W (2014). The ABC-F protein EttA gates ribosome entry into the translation elongation cycle. Nat Struct Mol Biol.

[10] Chen B, Boel G, Hashem Y (2014). EttA regulates translation by binding the ribosomal E site and restricting ribosome-tRNA dynamics. Nat Struct Mol Biol.

[11] Monk IR, Shah IM, Xu M, Tan M-W, Foster TJ (2012). Transforming the untransformable: application of direct transformation to manipulate genetically Staphylococcus aureus and *Staphylococcus epidermidis*. MBio.

[12] Fey PD, Endres JL, Yajjala VK (2013). A genetic resource for rapid and comprehensive phenotype screening of nonessential Staphylococcus aureus genes. MBio..

[13] Chaudhuri RR, Allen AG, Owen PJ (2009). Comprehensive identification of essential Staphylococcus aureus genes using transposon-mediated differential hybridisation (TMDH). BMC Genomics.

[14] Mäder U, Nicolas P, Depke M (2016). Staphylococcus aureus Transcriptome architecture: from laboratory to infection-mimicking conditions. PLoS Genet.

